# Antithrombotic Therapy Optimization in Patients with Atrial Fibrillation Undergoing Percutaneous Coronary Intervention

**DOI:** 10.3390/jcm13010098

**Published:** 2023-12-23

**Authors:** Felice Gragnano, Antonio Capolongo, Antonio Micari, Francesco Costa, Victoria Garcia-Ruiz, Vincenzo De Sio, Fabrizia Terracciano, Arturo Cesaro, Elisabetta Moscarella, Silvio Coletta, Pasquale Raucci, Fabio Fimiani, Leonardo De Luca, Giuseppe Gargiulo, Giuseppe Andò, Paolo Calabrò

**Affiliations:** 1 Department of Translational Medical Sciences, University of Campania “Luigi Vanvitelli”, 81100 Caserta, Italy; felice.gragnano@unicampania.it (F.G.); anto.capolongo@libero.it (A.C.); dott.vincenzodesio@gmail.com (V.D.S.); fabrizia.terracciano@gmail.com (F.T.); arturo.cesaro@unicampania.it (A.C.); elisabetta.moscarella@unicampania.it (E.M.); 2 Division of Clinical Cardiology, Azienda Ospedaliera di Rilievo Nazionale “Sant’Anna e San Sebastiano”, 81100 Caserta, Italy; silcoletta@gmail.com; 3 Department of Biomedical and Dental Sciences and Morphological and Functional Imaging, University of Messina, 98122 Messina, Italy; antonio.micari@unime.it (A.M.); dottfrancescocosta@gmail.com (F.C.); 4 Department of Cardiology, GIOMI Hospital, 98122 Messina, Italy; mavigaru@gmail.com; 5 Division of Health Technology Assessment, Azienda Ospedaliera di Rilievo Nazionale “Sant’Anna e San Sebastiano”, 81100 Caserta, Italy; rauccipasq@gmail.com; 6 Unit of Inherited and Rare Cardiovascular Diseases, Azienda Ospedaliera di Rilievo Nazionale Dei Colli, “Vincenzo Monaldi”, CCMR Regione Campania, 80138 Naples, Italy; fimianifabio@hotmail.it; 7 Division of Cardiology, Department of Cardiosciences, Azienda Ospedaliera San Camillo-Forlanini, 00152 Roma, Italy; leo.deluca@libero.it; 8 Department of Advanced Biomedical Sciences, University of Naples Federico II, 80138 Naples, Italy; peppegar83@libero.it; 9 Department of Clinical and Experimental Medicine, University of Messina, 98122 Messina, Italy; gando@unime.it

**Keywords:** DAPT, triple antithrombotic therapy, P2Y12 inhibitors, atrial fibrillation, PCI, bleeding

## Abstract

The antithrombotic management of patients with atrial fibrillation (AF) undergoing percutaneous coronary intervention (PCI) poses numerous challenges. Triple antithrombotic therapy (TAT), which combines dual antiplatelet therapy (DAPT) with oral anticoagulation (OAC), provides anti-ischemic protection but increases the risk of bleeding. Therefore, TAT is generally limited to a short phase (1 week) after PCI, followed by aspirin withdrawal and continuation of 6–12 months of dual antithrombotic therapy (DAT), comprising OAC plus clopidogrel, followed by OAC alone. This pharmacological approach has been shown to mitigate bleeding risk while preserving adequate anti-ischemic efficacy. However, the decision-making process remains complex in elderly patients and those with co-morbidities, significantly influencing ischemic and bleeding risk. In this review, we discuss the available evidence in this area from randomized clinical trials and meta-analyses for post-procedural antithrombotic therapies in patients with non-valvular AF undergoing PCI.

## 1. Introduction

Oral anticoagulation (OAC) therapy is recommended in several clinical conditions, with atrial fibrillation (AF) being the most common. Nowadays, more than 4 million people in Europe and 2 million in the U.S.A. suffer from AF [1]. As many as 20–30% of patients with AF undergo PCI, while approximately 10% of PCI candidates have chronic or new-onset AF, requiring long-term OAC [2,3]. Patients with AF usually require concomitant antiplatelet therapy following acute coronary syndrome (ACS) or percutaneous coronary intervention (PCI). This clinical scenario represents a challenge for antithrombotic management due to the simultaneous need to prevent coronary thrombotic events, along with cerebrovascular and systemic embolism, while minimizing the risk of bleeding. In this review, we discuss the available evidence from randomized controlled trials and meta-analyses for post-procedural antithrombotic strategies in patients with non-valvular AF undergoing PCI. The management of patients with valvular AF (i.e., with moderate to severe mitral stenosis or mechanical prosthetic valve) is beyond the scope of this article.

## 2. Antithrombotic Therapy in AF-PCI Patients: A Clinical Conundrum

**Figure 1:**
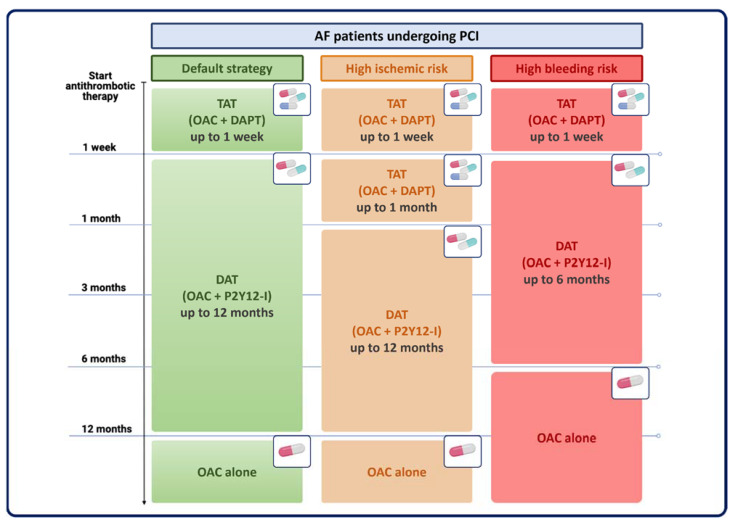
Antithrombotic strategies in AF-PCI patients according to individual ischemic and bleeding risk. AF = atrial fibrillation; DAPT = dual antiplatelet therapy; DAT = dual antithrombotic therapy; OAC = oral anticoagulation therapy; P2Y12-I = P2Y_12_ inhibitors; PCI = percutaneous coronary intervention; TAT = triple antithrombotic therapy.

Prevention of coronary ischemic events after PCI can be effectively achieved with dual antiplatelet therapy (DAPT) with aspirin and a P2Y12 inhibitor [4,5,6], while long-term use of OAC provides protection against cerebrovascular and systemic embolism in patients with AF [2]. The combination of DAPT plus OAC, the so-called triple antithrombotic therapy (TAT), is effective in reducing atherothrombotic and cardioembolic risk. However, it has been associated with up to a 3-fold increase in bleeding complications compared with less intensive regimens [7,8,9]. The hemorrhagic risk associated with TAT depends on its duration and type, and both can be modulated to improve clinical outcomes. In most cases, TAT duration should be limited to the early (1 week) post-PCI phase, while 1-month TAT could be considered in selected patients with high ischemic and low bleeding risk. Direct oral anticoagulants (DOACs) have a better safety profile than vitamin K antagonists (VKAs) and should be preferred as a first-line strategy (Figure 1). For concomitant antiplatelet therapy, early cessation of aspirin and continuation of the P2Y12 inhibitor clopidogrel plus OAC for 6–12 months is usually recommended, followed by OAC alone as a long-term maintenance strategy. Of note, the use of more potent agents, ticagrelor and prasugrel, in this setting is discouraged due to excessive bleeding hazards, and, therefore, they should be reserved for very selected cases. The above considerations summarize the results of several randomized trials conducted to determine the optimal pharmacological treatment of AF-PCI patients. Evidence from individual trials and subsequent study-level meta-analyses have been incorporated into current guidelines to provide practical algorithms for treatment decisions.

## 3. Randomized Trials Comparing Different Antithrombotic Regimens in AF-PCI Patients

Several randomized trials have compared prolonged TAT regimens with less intensive antithrombotic strategies in patients with AF undergoing PCI (Table 1). Early studies included VKA-based anticoagulation in the experimental and control groups, thus essentially reflecting the comparison of single versus dual antiplatelet therapy in addition to VKAs [10]. After the introduction of DOACs, three randomized trials [11,12,13] were designed to compare dual antithrombotic therapy (DAT) with a DOAC (i.e., rivaroxaban, dabigatran, or edoxaban) versus TAT with a VKA. One trial, given its factorial design, compared both anticoagulation regimens (apixaban versus VKAs) and antiplatelet regimens (aspirin versus placebo) to provide definitive answers on whether the type and/or duration of TAT are relevant for practice [14,15]. Importantly, in all four randomized DOAC trials [11,12,13,15], DAT consisted of OAC plus a P2Y_12_ inhibitor (mostly clopidogrel) with early aspirin discontinuation after ACS or PCI, a combination regimen that was considered the most promising in this setting based on pharmacological and clinical considerations [11,12,13,15]. In this area, a recent sub-analysis of a randomized trial also provided additional evidence evaluating an abbreviated versus standard antiplatelet regimen in high bleeding risk (HBR) patients undergoing PCI with an indication for OAC [16,17]. Finally, two randomized trials evaluated the safety and efficacy of OAC with or without single antiplatelet therapy in the long-term (>1 year) after PCI.

### 3.1. Randomized Trials of Antithrombotic Strategies within One Year after PCI

The WOEST trial [10] (What is the Optimal antiplatElet and Anticoagulant Therapy in Patients With Oral Anticoagulation and Coronary StenTing) randomized 573 PCI patients on OAC for AF (69%) or other medical conditions (31%) to receive clopidogrel alone (DAT group) or clopidogrel plus aspirin (TAT group) using an open-label design. The primary outcome of any bleeding episode within one year post-PCI occurred in 19.4% of patients receiving DAT and in 44.4% of those receiving TAT (HR: 0.36; 95% CI: 0.26–0.50; *p* < 0.0001). DAT was also associated with a lower incidence of the composite secondary endpoint of death, myocardial infarction (MI), stroke, target-vessel revascularization, and stent thrombosis compared to TAT (HR: 0.60; 95% CI: 0.38–0-94; *p* = 0.025).

The ISAR-TRIPLE [18] (Intracoronary Stenting and Antithrombotic Regimen-Testing of a 6-Week Versus a 6-Month Clopidogrel Treatment Regimen in Patients With Concomitant Aspirin and Oral Anticoagulant Therapy Following Drug-Eluting Stenting) trial was designed to evaluate whether reducing the duration of TAT from six months to six weeks by discontinuing clopidogrel in patients receiving concomitant aspirin and OAC after PCI with a drug-eluting stent (DES), improved the net clinical outcome of death, MI, definite stent thrombosis, stroke, or major bleeding at nine months. The trial randomized 614 patients to 6-week TAT versus 6-month TAT and showed no significant difference between the two treatment strategies in terms of the primary net clinical outcome (HR: 1.14; 95% CI: 0.68–1.91; *p* = 0.63). Similarly, no difference was observed in the key efficacy endpoint of cardiac death, MI, definite stent thrombosis, and ischemic stroke or the key safety endpoint of major bleeding.

Starting the DOAC era, the PIONEER-AF PCI [13] (Open-Label, Randomized, Controlled, Multicenter Study Exploring Two Treatment Strategies of Rivaroxaban and a Dose-Adjusted Oral Vitamin K Antagonist Treatment Strategy in Subjects with Atrial Fibrillation who Undergo Percutaneous Coronary Intervention) trial randomized 2124 patients with non-valvular AF undergoing PCI into three groups: rivaroxaban 15 mg once daily plus a P2Y12 inhibitor for 12 months (group 1); rivaroxaban 2.5 mg twice daily plus DAPT for 1, 6, or 12 months (group 2); standard therapy with a VKA plus DAPT for 1, 6, or 12 months (group 3). Rivaroxaban 15 mg or 2.5 mg plus a P2Y12 inhibitor for 12 months was associated with lower rates of clinically significant bleeding (16.8% in group 1, 18.0% in group 2, and 26.7% in group 3; HR for group 1 vs. group 3: 0.59, 95% CI: 0.47–0.76, *p* < 0.001; HR for group 2 vs. group 3: 0.63; 95% CI: 0.50–0.80, *p* < 0.001) with similar efficacy in terms of major adverse cardiovascular events (6.5% in group 1, 5.6% in group 2, and 6.0% in group 3; *p* values were not significant for all comparisons).

In the RE-DUAL PCI [12] (Randomised Evaluation of Dual Antithrombotic Therapy with Dabigatran versus Triple Therapy with Warfarin in Patients with Nonvalvular Atrial Fibrillation Undergoing Percutaneous Coronary Intervention) trial, 2725 patients with AF-PCI were randomized to receive TAT with warfarin plus clopidogrel or ticagrelor and aspirin for 1–3 months or DAT with clopidogrel or ticagrelor plus dabigatran 110 mg or 150 mg twice daily. The incidence of the primary endpoint (major or clinically relevant non-major bleeding event, defined by the International Society on Thrombosis and Hemostasis (ISTH)) was 15.4% in the DAT group with dabigatran 110 mg compared to 26.9% in the TAT group (HR: 0.52; 95% CI: 0.42–0.63; *p* < 0.001 for non-inferiority; *p* < 0.001 for superiority) and 20.2% in the DAT group with dabigatran 150 mg compared to 25.7% in the TAT group (HR: 0.72; 95% CI: 0.58–0.88; *p* < 0.001 for non-inferiority). In addition, DAT was not inferior to TAT for preventing thromboembolic events (HR: 1.04; 95% CI: 0.84–1.29; *p* = 0.005 for non-inferiority).

The ENTRUST-AF PCI [11] (Edoxaban Treatment Versus Vitamin K Antagonist in Patients With Atrial Fibrillation Undergoing Percutaneous Coronary Intervention) trial randomized 1506 AF-PCI patients to DAT with edoxaban 60 mg once daily plus a P2Y12 inhibitor for 12 months or TAT with a VKA for 1–12 months. The edoxaban-based regimen was not inferior to the VKA-based regimen regarding bleeding (HR: 0.83; 95% CI: 0.65–1.05; *p* = 0.001 for non-inferiority), with no significant differences in ischemic events.

In the AUGUSTUS trial [15] (An Open-label, 2-by-2 Factorial, Randomized Controlled Clinical Trial to Evaluate the Safety of Apixaban vs. Vitamin K Antagonist and Aspirin vs. Placebo in Patients With Atrial Fibrillation and Acute Coronary Syndrome and/or Percutaneous Coronary Intervention), the only DOAC trial with a 2-by-2 factorial design, 4614 patients were randomized to receive apixaban or a VKA plus aspirin or placebo for six months. Major and clinically relevant bleeding events were observed in 10.5% of the patients receiving apixaban compared to 14.7% of those receiving VKA (HR: 0.69; 95% CI: 0.58–0.81; *p* < 0.001 for non-inferiority and superiority) and in 16.1% of patients receiving aspirin compared to 9.0% of those receiving placebo (HR: 1.89; 95% CI: 1.59–2.24; *p* < 0.001). Patients on apixaban had a lower incidence of death or hospitalization than those in the VKA group (23.5% vs. 27.4%; HR: 0.83; 95% CI: 0.74–0.93; *p* = 0.002) and a similar incidence of ischemic events. In summary, the antithrombotic regimen with apixaban without aspirin resulted in less bleeding and fewer hospitalizations, with no significant differences in the incidence of ischemic events, compared with regimens with a VKA, aspirin, or both.

In the MASTER DAPT trial [16] (Management of High Bleeding Risk Patients Post Bioresorbable Polymer Coated Stent Implantation with an Abbreviated Versus Standard DAPT Regimen), 4579 patients at HBR were randomized after 1-month DAPT to abbreviated or standard antiplatelet therapy. Randomization was stratified by concomitant OAC indication. In the population with an OAC indication (N = 1666), at one month from PCI, patients changed immediately to single antiplatelet for five months (abbreviated regimen) or continued ≥ 2 months of dual antiplatelet and single antiplatelet (standard regimen). No difference was observed for the co-primary endpoints of net adverse clinical outcomes of death, MI, stroke, or Bleeding Academic Research Consortium (BARC) 3 or 5 bleeding (HR: 0.83; 95% CI: 0.60–1.15); nor were differences observed in the major adverse cardiac and cerebral events (MACCE) of death, MI, or stroke (HR: 0.88; 95% CI: 0.60–1.30), and BARC type 2, 3, or 5 bleeding (HR: 0.83; 95% CI: 0.62–1.12) between abbreviated versus standard antiplatelet regimens in addition to long-term OAC. Of note, in the per-protocol analysis of the trial, including the MASTER DAPT adherent OAC population, discontinuation of single antiplatelet therapy six months after PCI was associated with similar MACCE and lower BARC type 2, 3, or 5 bleeding (HR: 0.47; 95% CI: 0.22–0.99) than single antiplatelet therapy continuation, suggesting the potential benefit of this strategy [22].

Several issues should be considered when interpreting these findings. None of the trials discussed above were powered to draw definitive conclusions about the anti-ischemic efficacy of DAT versus TAT, especially for relatively rare events, such as stent thrombosis [7,13,18]. In all DOAC trials, aspirin was not discontinued at the time of PCI; therefore, patients in the DAT groups also received TAT after PCI for variable periods (up to 72 h in PIONEER-AF PCI, 120 h in RE-DUAL PCI, 14 days in AUGUSTUS, and 5 days in ENTRUST-AF PCI). There is also no conclusive evidence on whether aspirin or clopidogrel should be part of the DAT, and data on potent P2Y12 inhibitors are limited [4,12]. Finally, it should be noted that the evidence in high-risk patients, including those undergoing complex PCI or presenting with ACS, is derived from subgroup analyses of randomized trials and remains exploratory. In particular, specific subsets, such as patients with ST-segment elevation MI, were underrepresented in these trials (i.e., less than 10% of the trial populations); therefore, caution should be exercised when extending these results to these patients.

### 3.2. Randomized Trials of Antithrombotic Strategies beyond One Year after PCI

The OAC-ALONE trial [19] (Optimizing Antithrombotic Care in Patients With AtriaL fibrillatiON and Coronary stEnt) was designed to compare a single-drug strategy of OAC alone with a combination strategy of OAC plus single antiplatelet agent (aspirin or clopidogrel) in patients with AF from one year after stent implantation onwards. The study initially planned to enroll 2000 patients over 12 months but was terminated early after enrolling 696 patients over 38 months. The primary endpoint of all-cause death, MI, stroke, or systemic embolism was observed in 15.7% of patients treated with OAC alone and 13.6% of patients treated with OAC plus a single antiplatelet agent, not achieving the non-inferiority for the ischemic endpoint (HR: 1.16; 95% CI: 0.79–1.72; *p* = 0.20 for non-inferiority, *p* = 0.45 for superiority). The key secondary endpoint of all individual ischemic endpoints and ISTH major bleedings occurred in 19.5% of OAC-alone patients and 19.4% of those receiving OAC plus an antiplatelet agent (HR: 0.99; 95% CI: 0.71–1.39; *p* = 0.016 for non-inferiority, *p* = 0.96 for superiority). Overall, the trial was underpowered and inconclusive regarding the comparative efficacy and safety of the two strategies.

More recently, the AFIRE [20] (Atrial Fibrillation and Ischemic Events with Rivaroxaban in Patients With Stable Coronary Artery Disease Study) trial included 2236 patients with AF and established chronic coronary disease who had undergone PCI (1564 patients, 71.4% of whom with at least one DES) or coronary artery bypass grafting (252 patients, 11.4%) more than one year earlier or were managed medically. Patients were randomized to receive rivaroxaban monotherapy (15 mg daily for patients with a creatinine clearance of ≥50 mL/min, 10 mg daily for those with a creatinine clearance of 15–49 mL/min) or combination therapy with rivaroxaban plus a single antiplatelet agent. Rivaroxaban monotherapy was non-inferior to combination therapy for the primary efficacy endpoint of death, stroke, systemic embolism, MI, or unstable angina requiring revascularization (HR: 0.72; 95% CI: 0.55–0.95; *p* < 0.001 for non-inferiority) and was superior for the primary safety endpoint of major bleeding (HR: 0.59; 95% CI: 0.39–0.89; *p* = 0.01 for superiority). In the subgroup analysis by revascularization strategy, the point estimate of the treatment effect for the primary endpoint was numerically in favor of rivaroxaban monotherapy in the PCI subgroup (HR: 0.62, 95% CI: 0.45–0.85) but not in the CABG subgroup (HR: 1.19; 95% CI: 0.67–2.11). The trial was stopped early due to increased mortality with the combination therapy, and its results support current recommendations to discontinue antiplatelet therapy at 12 months after PCI and continue with OAC monotherapy. Of note, the inclusion of only East Asian patients and the Japanese-approved dose of rivaroxaban (10 mg or 15 mg once daily, depending on creatinine clearance) instead of the globally approved daily dose of 20 mg should be considered when interpreting these results.

## 4. Synthesis of Evidence from Meta-Analyses

Pivotal AF-PCI trials, each with a relatively small sample size distributed across multiple treatment arms, were generally designed to detect superiority for bleeding events and non-inferiority for ischemic events [11,12,13,15]. However, none had enough statistical power to explore rare events such as stent thrombosis or intracranial bleeding. To this purpose, meta-analyses are useful to enhance statistical power and more accurately determine the clinical impact of multiple antithrombotic treatment strategies on such uncommon events.

Given the immense complexity of treatment decisions for AF patients undergoing PCI or with ACS—considering antithrombotic type, duration, and dosage, which could result in hundreds of thousands of possible treatment permutations—it’s unsurprising that numerous articles have been published [23]. Despite only seven randomized trials investigating the impact of DAT or TAT in these patients [10,11,12,13,15,18,24], over 80 meta-analyses have been published. This abundance of meta-analyses arises because attempts to summarize evidence have been associated with various interpretations of data from the available trials. Specifically, the main differences in results from these meta-analyses are linked to diverse approaches to study inclusion, endpoint selection, timing, and treatment type and dosage. These are summarized in Table 2. In general, all published meta-analyses confirm original findings from individual trials that DAT is associated with a reduction of major or clinically relevant non-major bleeding compared with TAT. Golwala et al., in one of the first studies published in the field, confirmed that DAT, compared with TAT, was associated with a 47% reduction of TIMI (Thrombolysis in Myocardial Infarction) major and minor bleeding, maintaining a substantial equipoise in terms of trial-defined MACE [25]. This meta-analysis published in 2018 lacked the more definitive evidence provided by two additional pivotal AF-PCI trials, AUGUSTUS and ENTRUST-AF-PCI, which were available later. An updated meta-analysis by Gargiulo et al. [26] evaluated the impact of DAT compared to TAT, including all four pivotal AF-PCI trials, and encompassed 10,234 patients. In this study, DAT was associated with a 44% reduction of ISTH major or clinically relevant non-major bleeding and a 46% reduction of ISTH major bleeding [26]. Regarding ischemic endpoints, while prior meta-analyses confirmed no difference between DAT and TAT in terms of MACE, Gargiulo et al. suggested a possible increase of ischemic events in patients assigned to DAT, with a 59% increase in trial-defined stent thrombosis and a borderline increase in MI [26]. This was also confirmed by Andò et al., who found that DAT was associated with a significant 54% increase in stent thrombosis, a 23% increase in MI, a significant increase in cardiovascular mortality without impacting all-cause mortality or study-defined MACE [27]. The signal for higher rates of ischemic events with DAT was primarily driven by an excess of ischemic events in patients assigned to DAT with dabigatran 110 mg. A dedicated analysis of the AUGUSTUS trial also highlighted a possible increase in stent-related events in the first 30 days after the procedure in patients assigned to DAT [28]. This underscores, for the first time, the presence of a possible trade-off between ischemic and bleeding events to be considered in patients assigned to DAT or TAT. Notably, while a bleeding-ischemic event trade-off was observed, overall rates of bleeding events largely surpassed those of ischemic events, reflecting a lower number needed to treat with DAT to obtain a bleeding benefit compared to preventing rarer events such as stent thrombosis or MI. In this context, several elements have been suggested by international guidelines to base treatment decisions in these cases [29,30,31]. Clinical presentation has been considered a possible element to highlight a higher ischemic risk population that might benefit from a longer initial treatment with TAT. Therefore, Gargiulo et al. specifically explored the impact of DAT compared to TAT in 10,193 patients with ACS or CCS [32]. They found that irrespective of clinical presentation, DAT was associated with a similar reduction of ISTH major or clinically relevant non-major bleeding in both patients with ACS and CCS, where a reduction of 37% and 32%, respectively, was observed, with negative interaction testing [32]. In both subgroups, there was no difference between DAT and TAT for all-cause death, MACE, or stroke. However, MI and stent thrombosis were numerically higher with DAT versus TAT consistently in ACS and CCS [32].

**Figure 2:**
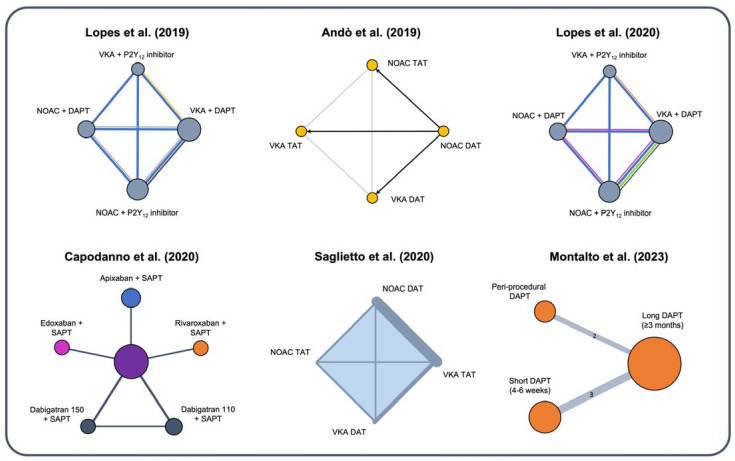
Different designs of recent network meta-analyses evaluating optimal treatment in AF patients with ACS or undergoing PCI [27,33,34,35,36,37]. DAPT = dual antiplatelet therapy; DAT = dual antithrombotic therapy; NOAC = novel oral anticoagulants; SAPT = single antiplatelet therapy; TAT = triple antithrombotic therapy; VKA = vitamin K antagonist.

Several network meta-analyses attempted to indirectly compare various treatment options with diverse designs (Figure 2) [27,33,34,35,36,37]. Lopes et al. included five randomized trials and 11,542 patients based on four different treatment types: NOAC + DAPT, NOAC + SAPT, VKA + DAPT, and VKA + SAPT [34]. They found that compared with VKA + DAPT, regimens of VKA-based and NOAC-based DAT were associated with a 43% and 48% reduction in TIMI major bleeding, respectively. All four treatments explored showed a similar risk of MACE [34]. Similarly, Saglietto et al. showed that both VKA and NOAC-based DAT regimens reduced the occurrence of TIMI major bleeding by 38% and 48%, respectively, but only NOAC-based DAT significantly reduced ICH by 67% compared to VKA-based TAT [35]. No difference in MACE was observed among the different explored treatment strategies, and a trend toward higher risks of ST was observed for NOAC-based DAT compared to both VKA and NOAC-based TAT. Capodanno et al. performed a network meta-analysis by including four trials, while the multiple treatment arms accounted for the different types of OAC associated with DAT or TAT [36]. They consistently found that NOAC-based DAT was associated with a 44% reduction of clinically significant bleeding. Interestingly, in this analysis, indirect comparisons taking into account the possible impact of different OAC types on overall safety performance showed that the safety profile of NOAC-based DAT might be affected by the type of OAC implemented, with a signal towards the highest safety of an apixaban-based DAT [36]. These studies confirmed that NOACs provide a more beneficial safety profile than VKA in an AF-PCI population and that a NOAC-based DAT strategy should be preferred. Nevertheless, the possible trade-offs in specific patients with a higher risk for stent-related events should be accounted for.

Importantly, most trials, and consequently the resulting evidence from meta-analyses, predominantly tie the safety and efficacy impact of DAT to NOAC therapy compared to a VKA-based TAT. As VKA is no longer the standard of care in AF-PCI patients and is no longer an informative comparator, it is crucial to evaluate the impact of DAT vs. TAT by unlinking OAC from the antiplatelet therapy regimen. Indeed, it is well-established that NOACs are associated with a reduced risk of bleeding per se compared to VKA, which could confound the impact of DAT vs. TAT irrespective of the antiplatelet regimen implemented [38]. To address this specific question, a recent meta-analysis by Montalto et al. explored the impact of DAPT and its duration, irrespective of the type of OAC, specifically excluding all trials that tied antiplatelet and anticoagulant strategy together [37]. Specifically, this meta-analysis aimed to determine the optimal duration of DAPT after PCI in patients with any indication for OAC. The study included five randomized clinical trials (N = 7665) that exclusively randomized patients to DAPT duration after PCI and excluded studies that tied the randomization process of DAT vs. TAT to OAC type [37]. The results suggest that abbreviated DAPT (i.e., periprocedural or up to 6 weeks) compared to prolonged DAPT (3 months or longer) in association with OAC is associated with a significant reduction of major or clinically relevant non-major bleeding and major bleeding (RR: 0.69; 95% CI: 0.52–0.91; *p* = 0.01 and RR: 0.70; 95% CI: 0.52–0.95; *p* = 0.01, respectively) with no difference for MACE (RR: 0.96; 95% CI: 0.70–1.33; *p* = 0.6), all-cause death, cardiovascular death, stent thrombosis, or MI [37]. A network meta-analysis comparing three different treatment strategies, namely peri-procedural TAT, short TAT for 4–6 weeks, and longer TAT for ≥3 months, showed that peri-procedural TAT had the highest probability of preventing clinically relevant non-major bleeding and major bleeding, while still having the highest probability of ranking better for MACE compared to the other two treatment strategies [37].

## 5. Ischemic and Bleeding Risk Stratification in Patients with AF Undergoing PCI

Assessment of ischemic and bleeding risk is critical to inform the clinical decision-making in patients with AF undergoing PCI. The presence (or absence) of ischemic risk factors and additional bleeding risk determinants beyond OAC per se should be considered to individualize their management with respect to antithrombotic therapy (and more).

To stratify ischemic risk in patients undergoing PCI, the recent 2023 European Society of Cardiology (ESC) guidelines for the management of ACS [39] propose several high-risk features for stent-driven recurrent events, including clinical characteristics (i.e., chronic kidney disease, prior stent thrombosis on antiplatelet therapy), coronary anatomy (i.e., multivessel disease, complex coronary lesions) and procedural features (i.e., at least three stents implanted, at least three lesions treated, bifurcation with two stents implanted, stenting of the last remaining patent coronary artery, total stent length > 60 mm, treatment of a chronic total occlusion) [40]. This stratification approach is similar to previous guidelines and consensus documents, including the 2020 ESC guidelines on AF [7], which also included patient-related and procedure-related risk factors to identify AF-PCI patients at increased risk of recurrent ischemic events. While these risk criteria are useful to stratify ischemic risk, whether they can assist clinicians in deciding the type and duration of TAT and DAT remains unclear. Interestingly, a recent post-hoc analysis of the RE-DUAL PCI trial developed and validated a novel risk score to identify AF-PCI patients at increased risk of thrombotic events who may benefit from prolonged TAT over DAT during the first year after PCI [31]. Six clinical variables (namely, left ventricular ejection fraction, 3-vessel disease, MI at presentation, history of peripheral arterial disease, platelet count ≥ 400 × 10^9^/L, and eGFR ≥ 90 mL/min) were selected to predict the occurrence of thrombotic events (defined as cardiovascular death, MI, stent thrombosis or ischemic stroke). In patients at low or intermediate risk (score < 5), the use of TAT was significantly associated with higher bleeding rates than DAT (25.6% vs. 15.1%; *p* < 0.001), with no clear benefit for ischemic protection. Conversely, in high-risk patients (score ≥ 5), the use of TAT was associated with significantly fewer MI and stent thrombosis (6.3% vs. 21.0%; *p* = 0.041) [31]. Therefore, this score may be useful to identify patients in whom the use of prolonged TAT (i.e., beyond one week) may provide a benefit in terms of ischemic events.

For bleeding risk assessment, several algorithms and risk scores have been developed in patients with AF or undergoing PCI, but prospective validation analyses focusing on AF-PCI patients remain limited. The Academic Research Consortium for High Bleeding Risk (ARC-HBR) criteria have recently been proposed, based on an expert consensus, to identify HBR patients undergoing PCI. This framework consists of major criteria (any criterion that, in isolation, confers a BARC 3 or 5 bleeding risk of ≥4% or is associated with a risk of intracranial hemorrhage of ≥1% at one year) and minor criteria (any criterion that, in isolation, confers an increased bleeding risk below the cut-offs for major criteria). In this framework, long-term OAC is considered a major criterion, and its impact on bleeding risk has been recently assessed in a large validation analysis of the ARC-HBR criteria, including 16,580 PCI patients. In this study, 452 patients receiving long-term OAC had a higher risk of BARC 3 or 5 bleeding compared to those with no ARC-HBR criteria, with an incidence rate that met the standard cut-off of 4% (1-year cumulative incidence: 2.52; 95% CI: 1.40–4.50). Of note, although the ARC-HBR definition was developed as a qualitative risk algorithm, its implementation as a point-based score in some validation analyses showed that the risk of bleeding increased proportionally as the number of ARC-HBR criteria increased, suggesting that in AF-PCI patients, the risk of bleeding is influenced by the presence of additional ARC-HBR criteria beyond OAC. These findings have also been validated in different subgroups of patients stratified by sex and clinical presentation, showing good predictive ability and discrimination performance [41,42].

The PARIS (Pattern of non-adherence to the antiplatelet regimen in stented patients) registry [43] included patients treated with drug-eluting stents. This registry identified two risk scores: one for thrombotic risk and one for hemorrhagic risk. For the second score, the predictor items were TAT at discharge, older age, BMI, anemia, smoking, and renal dysfunction.

The PRECISE-DAPT score was originally developed and validated for predicting bleeding in DAPT-treated PCI patients. Importantly, secondary validation analyses evaluated the score’s performance in patients receiving other antithrombotic regimens [44,45], including AF-PCI patients on long-term OAC. A sub-analysis of the RE-DUAL PCI trial examined the impact of bleeding risk based on the PRECISE-DAPT score on decision-making regarding DAT versus TAT [30]. PRECISE-DAPT was available in 2336 of 2725 participants, and 37.9% were defined as HBR (score ≥25 points). DAT with dabigatran 110 mg reduced bleeding compared to TAT in non-HBR patients (HR: 0.42; 95% CI: 0.31–0.57) and HBR patients (HR: 0.70; 95% CI: 0.52–0.94), with an effect that was more pronounced in non-HBR patients (*p*-interaction = 0.02). Similarly, DAT with dabigatran 150 mg reduced bleeding compared to TAT in non-HBR patients (HR: 0.60; 95% CI: 0.45–0.80), with a tendency to lower benefit in HBR patients (HR: 0.92; 95% CI: 0.63–1.34; *p*-interaction = 0.08). The risk of ischemic events was similar to DAT versus TAT in non-HBR and HBR patients, with non-significant interaction testing [30].

Other bleeding risk scores, including the HAS-BLED, CRUSADE, and ACUITY, have shown good predictive value for bleeding in patients with AF or PCI and are also recommended by current guidelines for risk stratification [46,47,48]. However, large validation analyses of these scores in AF-PCI patients receiving long-term OAC are currently lacking [46,47,48].

## 6. European and American Guidelines Recommendations

The 2023 ESC guidelines for the management of ACS recommend that the default strategy in AF-PCI with an acute presentation is TAT for one week, followed by DAT for 12 months, and then OAC alone (Class I). In addition, based on the available evidence, the guidelines support the use of DOACs over VKAs (due to the bleeding benefit) and clopidogrel as the antiplatelet agent of choice in DAT (as this agent was used in > 90% of patients in randomized trials). The guidelines also provide two alternative strategies for tailoring antithrombotic therapy according to patient risk. In patients in whom concerns about ischemic risk outweigh bleeding risk, TAT should be continued for one month (Class IIa). Conversely, in patients in whom concerns about bleeding risk predominate, discontinuation of DAT after six months and continuation of OAC alone may be considered (Class IIb). In addition, reduced doses of rivaroxaban (15 mg/day) or dabigatran (110 mg twice daily) should be considered to mitigate the risk of bleeding (Class IIa). Using prasugrel or ticagrelor as part of TAT or DAT is not recommended [7,39,49].

A similar default strategy (i.e., TAT of 1 week, DAT up to 12 months, and OAC alone thereafter) is recommended by the 2019 ESC guidelines for the management of chronic coronary syndrome [49]. Yet, some differences can be noted, specifically that TAT of ≥1 month and up to 6 months should be considered if the risk of stent thrombosis outweighs the risk of bleeding (Class IIa) and that DAT with ticagrelor or prasugrel may be considered as an alternative to TAT with OAC, aspirin, and clopidogrel in patients at moderate or high risk of stent thrombosis (Class IIb) [49].

In the 2020 ESC guidelines for the management of AF, the recommendations for antithrombotic therapy in patients with acute or chronic coronary syndromes largely overlap with those reported in the specific ESC documents described above (ref. 2019 CCS and 2023 ACS ESC Guidelines). Similar recommendations are also reported in the 2019 AHA/ACC guidelines for the management of AF [50] and in the 2021 AHA/ACC guidelines for coronary revascularization [51], which support as default strategy a short TAT period of 1–4 weeks followed by DAT for 6–12 months, and OAC as long-term monotherapy one year after revascularization. In line with the ESC guidelines, the American guidelines also support the preferred use of DOACs over VKAs because of their advantages in terms of bleeding prevention.

## 7. Ongoing Studies

Several clinical studies are currently ongoing. The OPTIMA-3 trial [21] will enroll approximately 2200 patients with ACS undergoing PCI and receiving OAC with warfarin. Patients will be randomized to an experimental arm with clopidogrel after 1-month DAPT and a control arm with clopidogrel plus aspirin for six months, followed by clopidogrel for up to 12 months for a primary composite endpoint of cardiovascular death, MI, ischemic stroke, systemic thromboembolism, and unplanned revascularization at 12 months; the major secondary endpoint is ISTH major bleeding or clinically relevant non-major bleeding. The OPTIMA-4 trial [21] will randomize approximately 1470 patients with ACS undergoing PCI to compare an antiplatelet therapy with clopidogrel versus ticagrelor on a background anticoagulation regimen of dabigatran 110 mg twice daily for a safety endpoint of major or nonmajor clinically significant bleeding and an efficacy endpoint of MACCE at 12 months (a composite of cardiovascular death, MI, ischemic stroke, systemic thromboembolism, and unplanned revascularization).

The MATRIX-2 (NCT05955365) and WOEST-3 (NCT04436978) trials are also underway to evaluate the safety and efficacy of antiplatelet-based strategies in the first month after PCI with delayed OAC therapy (e.g., beyond one month) compared to guideline-directed therapy in AF-PCI patients.

## 8. Conclusions

In AF patients with ACS or undergoing PCI, the conundrum of appropriate antithrombotic therapy remains. In most patients, TAT is currently recommended for a short period (i.e., one week), followed by clopidogrel plus OAC for up to 1 year, and OAC alone as long-term monotherapy. However, this strategy is not optimal for all patients, and tailored therapeutic approaches need to be considered in clinical practice, taking into account the ischemic and bleeding risk of each patient.

## Figures and Tables

**Table 1:** Randomized clinical trials including AF-PCI patients.

Trial	Year	No. of Patients	Study Population	Experimental Group	Control Group	Key Endpoints	Results
WOEST [10]	2013	573	OAC and PCI (ACS 27.1%)	OAC + P2Y_12_I (clopidogrel) for 1 to 12 months	OAC + P2Y_12_I (clopidogrel) + aspirin for 1 to 12 months	Any bleeding episode.	HR: 0.36; 95% CI: 0.26–0.50; *p* < 0.0001
ISAR-TRIPLE [18]	2015	614	OAC and PCI (ACS 32%)	OAC + P2Y_12_I (clopidogrel) + aspirin for 6 weeks	OAC + P2Y_12_I (clopidogrel) + aspirin for 6 months.	Composite of ischemic events (death, MI, definite stent thrombosis, stroke) and TIMI major bleeding.	HR: 1.14; 95% CI: 0.68–1.91; *p* = 0.63
PIONEER- AF PCI [13]	2016	2124	AF and PCI (ACS 51.6%)	OAC (rivaroxaban 15 mg/day) + P2Y_12_ I for 12 months (group 1); OAC (rivaroxaban 2.5 mg twice daily) + DAPT for 1, 6, or 12 months (group 2).	VKA + DAPT for 1, 6, or 12 months (group 3).	Clinically significant bleeding (composite of major or minor bleeding according to TIMI criteria, and bleeding requiring medical attention).	HR (group 1 vs. group 3): 0.59; 95% CI: 0.47–0.76; *p* < 0.001. HR (group 2 vs. group 3): 0.63; 95% CI: 0.50–0.80; *p* < 0.001.
RE-DUAL PCI [12]	2017	2725	AF and PCI (ACS 64%)	OAC (dabigatran 110 mg or 150 mg twice daily) + P2Y_12_ I (clopidogrel or ticagrelor)	Warfarin + DAPT (aspirin + clopidogrel or ticagrelor) for 1 month (BMS) or 3 months (DES)	Major or clinically relevant nonmajor bleeding event (ISTH criteria)	HR (dabigatran 110 mg b.i.d.): 0.52; 95% CI: 0.42–0.63; *p* < 0.001 for non-inferiority; *p* < 0.001 for superiority. HR (dabigatran 150 mg b.i.d.): 0.72; 95% CI: 0.58–0.88; *p* < 0.001 for non-inferiority; *p* = 0.002 for superiority
ENTRUST-AF PCI [11]	2019	1506	AF and PCI (ACS 52%)	OAC (edoxaban 60 mg) + P2Y_12_I for 12 months	OAC (VKA) + DAPT for 1–12 months	Major bleeding or clinically relevant nonmajor bleeding (ISTH criteria)	HR: 0.83; 95% CI: 0.65–1.05; *p* = 0.001 for non-inferiority; *p* = 0.1154 for superiority.
AUGUSTUS [15]	2019	4614	AF and PCI (ACS 61.2%)	OAC (apixaban 5 mg bid or VKA) + P2Y_12_I for 6 months	OAC (apixaban or VKA) + DAPT for 6 months	Major bleeding or bleeding clinically relevant nonmajor (ISTH criteria)	HR (apixaban vs. VKA): 0.69; 95% CI: 0.58–0.81; *p* < 0.001 for both non-inferiority and superiority. HR (aspirin vs. placebo): 1.89; 95% CI: 1.59–2.24; *p* < 0.001.
MASTER DAPT [16] (OAC sub-analysis)	2021	4579 (1666)	HBR and PCI after 1-month DAPT (OAC indication) (ACS 42.2%)	Abbreviated DAPT regimen (SAPT for 5 months + OAC)	Standard DAPT regimen (DAPT for 2 months + SAPT until 11 months + OAC)	First co-primary endpoint: NACE (death, MI, stroke, and BARC 3 or 5 bleeding) Second co-primary endpoint: MACCE (death, MI, or stroke) Third co-primary endpoint: major or clinically relevant nonmajor bleedings (BARC type 2, 3, or 5)	HR (NACE): 0.83; 95% CI; 0.60–1.15; *p* = 0.26. HR (MACCE): 0.88; 95% CI; 0.60–1.30. HR (BARC 2, 3 or 5): 0.83; 95% CI; 0.62–1.12; *p* = 0.25.
OAC-ALONE [19]	2019	696	AF beyond 1 year after PCI	OAC for 12 months	OAC + SAPT for 12 months	Primary endpoint: all-cause death, MI, stroke, or systemic embolism. Major secondary endpoint: primary endpoint or major bleeding (ISTH criteria).	HR (primary endpoint): 1.16; 95% CI: 0.79–1.72; *p* = 0.20 for non-inferiority, *p* = 0.45 for superiority. HR (major secondary endpoint): 0.99; 95% CI, 0.71–1.39; *p* = 0.016 for non-inferiority, *p* = 0.96 for superiority.
AFIRE [20]	2019	2236	AF and PCI or CABG (>1 year earlier) or CAD not requiring revascularization	OAC (rivaroxaban) for 6 months	OAC (rivaroxaban) + SAPT for 6 months	Primary efficacy endpoint: stroke, systemic embolism, MI, unstable angina requiring revascularization, or death from any cause. Primary safety endpoint: major bleeding (ISTH criteria).	HR (efficacy endpoint): 0.72; 95% CI: 0.55–0.95; *p* < 0.001 for non-inferiority. HR (safety endpoint): 0.59; 95% CI: 0.39–0.89; *p* = 0.01 for superiority.
OPTIMA-3 [21]	2024 (study completion estimated)	2274	AF and PCI (ACS 100%)	OAC (warfarin) + DAPT for 1 month, followed by SAPT (clopidogrel) up to 12 months	OAC (warfarin) + DAPT (clopidogrel + aspirin) for 6 months, followed by SAPT (clopidogrel) up to 12 months	Primary endpoint: MACCE at 12 months. Major secondary endpoint: major bleeding or bleeding clinically relevant nonmajor (ISTH criteria).	Ongoing
OPTIMA-4 [21]	2024 (study completion estimated)	1472	AF and PCI (ACS 100%)	OAC (dabigatran 110 mg twice daily) + SAPT (clopidogrel) up to 12 months	OAC (dabigatran 110 mg twice daily) + SAPT (ticagrelor) up to 12 months	Primary efficacy endpoint: MACCE at 12 months Primary safety endpoint: Major bleeding or bleeding clinically relevant nonmajor (ISTH criteria).	Ongoing

ACS = acute coronary syndrome; AF = atrial fibrillation; BARC= Bleeding Academic Research Consortium; BMS = bare metal stent; CABG = coronary Artery Bypass Graft; CAD = coronary artery disease; DAPT = dual antiplatelet therapy; DES = drug-eluting stent; HBR = high bleeding risk; ISTH = International Society on Thrombosis and Haemostasis; MACCE = major adverse cardiac and cerebrovascular event; MI = myocardial infarction; NACE = net adverse clinical events; OAC = oral anticoagulation therapy; P2Y_12_I = P2Y_12_ inhibitors; PCI = percutaneous coronary intervention; SAPT = single antiplatelet therapy; TIMI = Thrombolysis In Myocardial Infarction; VKA = vitamin K antagonists.

**Table 2:** Differential elements considered in AF-PCI meta-analyses.

Differential Elements Considered in AF-PCI Meta-Analyses
Inclusion or exclusion of WOEST trial: the inclusion of the WOEST trial in AF-PCI meta-analyses has been inconsistent. This is primarily due to its exclusive use of VKA and the inclusion of patients with various indications for long-term OAC beyond AF, such as mechanical heart valves and VTE.
Inclusion or exclusion of ISAR-TRIPLE trial: the inclusion of the ISAR-TRIPLE trial in AF-PCI meta-analyses has been inconsistent. This is primarily due to its exclusive use of VKA and the implementation of an initial 6-week period of TAT in both treatment arms post-study inclusion.
Inclusion or exclusion of SAFE-A trial: the inclusion of the SAFE-A trial in AF-PCI meta-analyses has been inconsistent, generally attributed to the initial 4-week period of TAT in both treatment arms post-study inclusion.
Inconsistent inclusion of ISAR-TRIPLE trial outcomes occurring before after randomization (landmark analysis).
Decision to merge or separate outcome data from the two DAT randomization arms in the RE-DUAL-AF-PCI trial, specifically Dabigatran 110 mg bid plus P2Y12I and Dabigatran 150 mg plus P2Y12I.
Decision to include or exclude the triple antithrombotic therapy arm with rivaroxaban 2.5 mg bid plus DAPT from the PIONEER-AF-PCI trial.
Decision to include or exclude the four treatment arms of the factorial randomization from the AUGUSTUS trial.
Decision to include or exclude patients with ACS not undergoing coronary stenting in the AUGUSTUS trial.

ACS = acute coronary syndrome; AF = atrial fibrillation; DAT = dual antithrombotic therapy; OAC = oral anticoagulation therapy; P2Y_12_I= P2Y_12_ inhibitors; PCI = percutaneous coronary intervention; TAT = triple antithrombotic therapy; VKA = vitamin K antagonists; VTE = venous thromboembolism.

## Data Availability

Not applicable.
